# Comparative analysis of 84 chloroplast genomes of *Tylosema esculentum* reveals two distinct cytotypes

**DOI:** 10.3389/fpls.2022.1025408

**Published:** 2023-01-31

**Authors:** Jin Li, Christopher Cullis

**Affiliations:** Department of Biology, Case Western Reserve University, Cleveland, OH, United States

**Keywords:** *Tylosema esculentum*, legume, chloroplast genome, genetic diversity, genomic structure, phylogenetic analysis, heteroplasmy, plastome evolution

## Abstract

*Tylosema esculentum* (marama bean) is an important orphan legume from southern Africa that has long been considered to have the potential to be domesticated as a crop. The chloroplast genomes of 84 marama samples collected from various geographical locations in Namibia and Pretoria were compared in this study. The cp genomes were analyzed for diversity, including SNPs, indels, structural alterations, and heteroplasmy. The marama cp genomes ranged in length from 161,537 bp to 161,580 bp and contained the same sets of genes, including 84 protein-coding genes, 37 tRNA genes, and 8 rRNA genes. The genes *rpoC2* and *rpoB*, and the intergenic spacers *trnT-trnL* and *ndhG-ndhI* were found to be more diverse than other regions of the marama plastome. 15 haplotypes were found to be divided into two groups, differing at 122 loci and at a 230 bp inversion. One type appears to have greater variability within the major genome present, and variations amongst individuals with this type of chloroplast genome seems to be distributed within specific geographic regions but with very limited sampling for some regions. However, deep sequencing has identified that within most of the individuals, both types of chloroplast genomes are present, albeit one is generally at a very low frequency. The inheritance of this complex of chloroplast genomes appears to be fairly constant, providing a conundrum of how the two genomes co-exist and are propagated through generations. The possible consequences for adaptation to the harsh environment in which *T. esculentum* survives are considered. The results pave the way for marama variety identification, as well as for understanding the origin and evolution of the bean.

## Introduction

*Tylosema esculentum*, also known as marama bean or gemsbok bean, is a perennial legume that naturally grows in the arid and semi-arid environments of Kalahari Desert, South Africa and surrounding areas (National Research Council, 1979). Marama beans have developed some special morphological and physiological characteristics, such as the use of giant tubers that store water to help them survive in harsh environments with prolonged drought and heat, where traditional crops cannot grow (Cullis et al., 2018; Lawlor, 2018; Cullis et al., 2022). Marama is used as a food source for local populations. Both its seeds and tubers are edible and nutritious, with seeds containing approximately 29-38% protein and 32-42% fat, comparable to many commercial crops like soybean and peanuts (Holse et al., 2010). In addition, marama also contains a variety of phytochemicals, including phenolics, flavonoids, saponins, and phytosterols, that help boost the immune system and help reduce the risk of cancer, diabetes, and cardiovascular diseases (Khan, 2018; Omotayo and Aremu, 2021). Because of these advantages, marama is also known as the green gold of Africa. However, the main source of acquisition for local villagers is still picking from wild plants. The domestication and breeding of marama has the potential to improve food security threatened by climate change and global warming in Southern Africa.

Marama is a non-nodulating legume from the subfamily Cercidoideae, which contains over 400 species in 14 genera (LPWG, 2017; Sinou et al., 2020). The genus *Tylosema*, in the tribe Cercideae subtribe Bauhiniinae, contains three other species, *Tylosema argentea*, *Tylosema fassoglense* and *Tylosema humifusa* (Coetzer and Ross, 1977). These are distributed throughout Africa, but marama is endemic to the semiarid regions of Southern Africa (Jackson et al., 2010; Wunderlin, 2010). However, even marama plants grown in the same environment and of the same age still exhibited very large differences in phenotypes such as plant size and vegetative growth rate (Cullis et al., 2019). Therefore, the selection of improved marama individuals and the study of the molecular genetic basis behind the phenotype of interest are of great significance for the breeding of the bean. In addition, marama also serves as a good model to explore the impact of extreme environments on plant evolution.

Chloroplasts are important organelles in plants, not only for photosynthesis, but also involved in regulating plant responses to stress conditions such as heat and drought (Estavillo et al., 2011; Song et al., 2021). The chloroplast genome is commonly configured as a double-stranded circular molecule. It consists of four parts, a small single-copy region (SSC) and a large single-copy region (LSC), separated by a pair of inverted repeats (IRa and IRb) with a small genomic size ranging from 120 to 170 kb (Palmer, 1991; Smith, 2017). The inverted repeats are thought to play an important role in maintaining chloroplast genome stability and conserved sequence arrangement (Palmer and Thompson, 1982). Chloroplast genome size is largely determined by the expansion, contraction and even loss of IR, which is particularly pronounced in legumes (Wang et al., 2018). Compared with nuclear DNA, plant chloroplast genomes are highly conserved with low recombination and substitution rate (Banks and Birky, 1985; Twyford and Ness, 2016). They generally contain 110-130 common genes (Jansen et al., 2005), so they are often used for phylogenetic analysis and species identification (Manos et al., 2008). Chloroplasts are semi-autonomous organelles (Dobrogojski et al., 2020), in which a large number of proteins are encoded by the nucleus. During the evolution of angiosperms, many chloroplast genes have been transferred into the nucleus, and in the original organelle genome, they are either lost or become pseudogenes (Martin, 2003). However, the remaining genes in cpDNA are thought to still play important roles in cellular activity (Drescher et al., 2000; Zhang et al., 2020).

Heteroplasmy, which refers to the coexistence of different types of organellar DNA in the same cell or individual, has previously been reported in Fabaceae (Johnson and Palmer, 1989; Lei et al., 2016; Lee et al., 2021). This could be caused by the accumulation of mutations in organellar DNA over time, or occasional non-Mendelian biparental cytoplasmic inheritance (Kondo et al., 1990; Carbonell-Caballero et al., 2015; Ramsey and Mandel, 2019; Iannello et al., 2021). In theory, this gives plants stronger adaptability allowing genotypes favored by natural selection to be retained. The organelle genetic bottleneck hypothesis suggests that allele frequencies may change rapidly during transmission from one generation to the next, possibly as a result of environmental influences (Ashley et al., 1989; Zhang et al., 2018). In other words, cells tend to pass on healthier, more adaptable organelles to offspring (Marlow, 2017). Occasionally, offspring with only mutant organellar DNA can be produced by a heteroplasmic mother. The gene *MSH1* has been found to play an important role in sorting chloroplast heteroplasmy, but this is mainly to correct for *de novo* mutations (Broz et al., 2022). Heteroplasmy arising from paternal leakage may have different fates. The study of heteroplasmy will contribute to a better understanding of organelle maintenance and inheritance in marama.

The genome size of marama bean was found to be small in legumes, about 1 Gb, calculated based on the size of the WGS data and the corresponding genome coverage (Cullis et al., 2019). Feulgen staining indicated that marama may be an ancient hexaploid plant with 44 chromosomes (Takundwa et al., 2012) although it could also be an ancient tetraploid since there are legumes with a haploid chromosome number of 11 (Bandel, 1974). An rDNA marker-based study has shown that marama has low inter-population diversity but high intra-population diversity, possibly due to the lack of gene flow between populations in the environment where marama grows, but the bean itself is a predominantly outcrossing plant (Nepolo et al., 2010). The chloroplast genome of marama was previously sequenced and assembled using a hybrid method based on both Illumina and PacBio datasets, with a total length of 161,537 bp (KX792933.1) (Kim and Cullis, 2017). Compared with the plastid genome of *Cercis canadensis*, a 7479 bp sequence containing the genes *rbcL*, *accD*, *psaI*, *ycf4*, *cemA*, and *petA* was inverted in the cp genome of marama, and this inversion was not seen in other legumes (Kim and Cullis, 2017). The same approach was also used to assemble the mitochondrial genome of marama, which contains a 9,798 bp long insertion of cpDNA with potentially non-functional chloroplast genes *psaA*, *psaB*, and *psbC* (Li and Cullis, 2021). This insertion has a large number of mutations compared to the original chloroplast sequence, and studying the base ratios at these loci helps to distinguish heteroplasmy in the chloroplast genome from differences in homologous sequences between different organelles in alignment.

**Figure 1:**
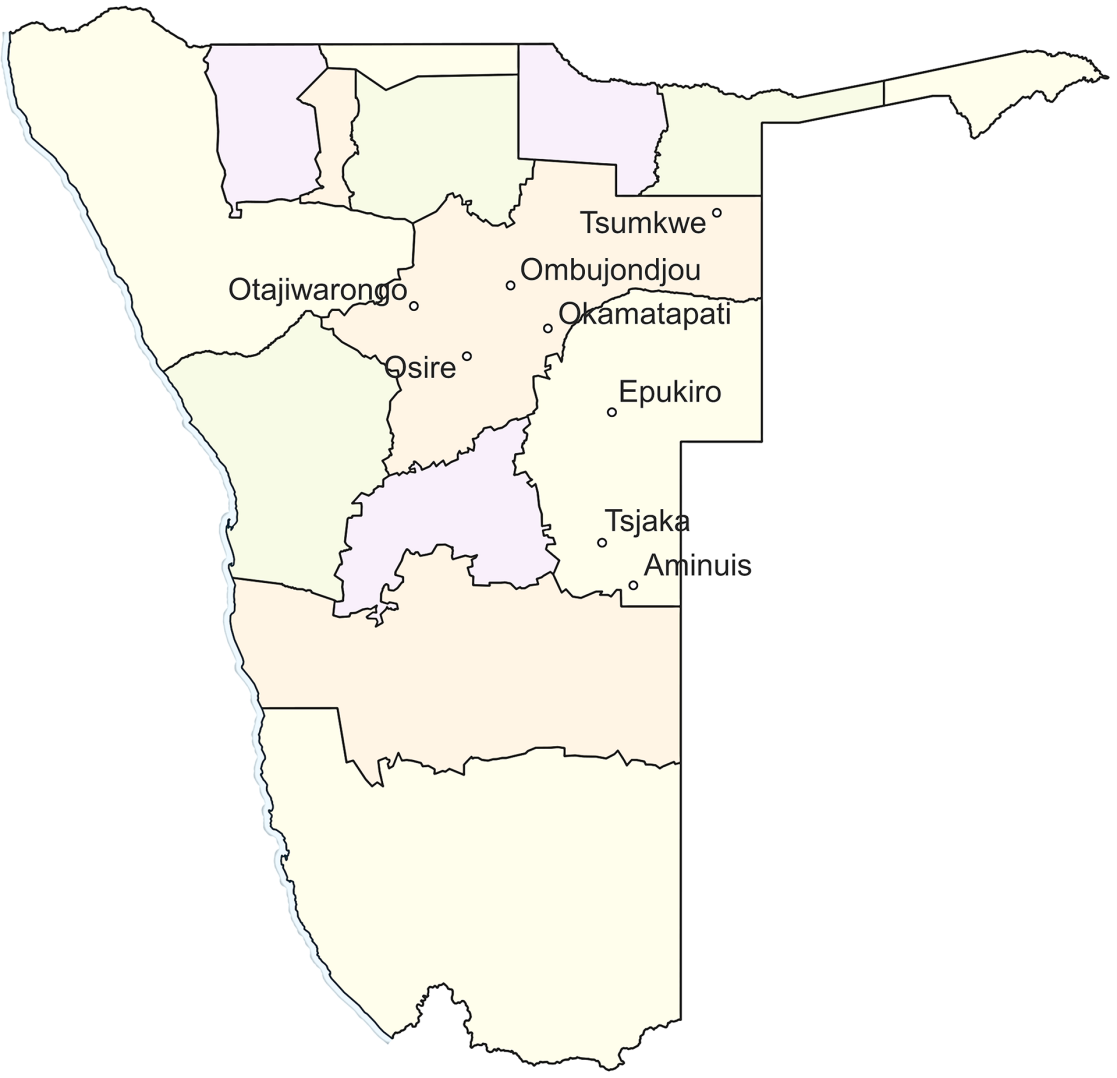
A map of Namibia showing the eight different locations where the wild marama samples were collected.

A comparative genomics study was performed in this study on the chloroplast genomes of 84 marama individuals collected from various geographic regions (**Figure 1**; **Table S1**) to identify polymorphisms including structural, single nucleotide and indels variants. This facilitates the study of phylogenetic relationships between the different marama populations and also between the individuals, which may help us better understand the origin of marama and the impact of extreme environments on plant genome evolution. In addition, SSRs in the plastid genome were analyzed and mutations that altered the coding sequence of the gene were reported, which could be useful in the breeding of the bean. Furthermore, plastid heteroplasmy and associated inheritance were investigated by analyzing chloroplast DNA allele frequencies and by comparing the chloroplast genomes of related plants.

## Materials and methods

### Plant materials

A total of 84 marama plants that were growing in South Africa and Namibia were sampled. Of these, 6 were plants that are growing on the University of Pretoria (UP) Farm (S25 45.490 E28 11.368) and 38 wild individuals from different geographical locations in Namibia, including Tsjaka (S22 75.039 E19 20.712), Okamatapati (S20 40.233 E18 21.59), Aminuis (S23 38.000 E19 22.00), Osire (S21 02.031 E17 21.244), Tsumkwe (S19 21.000 E20 16.000), Ombujondjou (S20 18.600 E17 58.525), Epukiro (S21 39.642 E19 25.092) and Otjiwarongo (S20 46.092 E16 65.123) (**Figure 1**). The remaining 40 were progeny plants grown from seeds collected from plants from Aminuis, a small holding in Namibia designated UNAM Farm and University of Pretoria Farm (**Table S1**). Note that although these two areas are designated as farms, marama is not being cultivated as a crop, but being deliberately maintained in these two curated locations as long-term perennial plants.

The 6 plants that are currently growing on the University of Pretoria (UP) farm were collected from North-West South Africa in the 1990’s but exact original location is not known. These plants differ phenotypically in a number of characteristics, including leaf size, internode length, stamen length and overall vigor (rate of stem elongation each growing season). DNA was extracted from leaves of the individual marama plants that were sampled from the various regions but not maintained as specimens. The progeny from individuals from the various regions do not have maternal data since at collection the pods were mature and all the foliage was dead, since the vegetative growth senesces each year. The plants from the Namibia small holding (designated UNAM Farm) were planted in 2010 from seed supplied by Professor P. Chimwamurombe but no identification of the origin of this seed was recorded. The DNA for the M samples (except M40) were extracted from immature seeds from a single plant for which DNA, leaf and flower stored material is available.

Therefore, the material sampled includes individual plants from various geographical locations as well as progeny from 7 different maternal individuals from various regions. Since it is expected that the chloroplast is maternally inherited, only one sample from each family is included in the analyzed data (43 samples), although full data is available for all 84 samples.

### DNA extraction and high-throughput sequencing

DNA was extracted from fresh young leaves or the embryonic axis of germinating seeds. The plant material was frozen in liquid nitrogen and ground with a mortar and pestle. Total genomic DNA was extracted using the QIAGEN DNeasy^®^ Plant Kit following the manufacturer’s protocol. The quality and quantity of the produced DNA were estimated by a NanoDrop™ 8000 Spectrophotometer and by electrophoresing on a 1.0% agarose TBE gel. The DNA was also quantified by the Qubit™ 3.0 Fluorometer after mixing 5 μL of DNA with 195 µL of Qubit^®^ working solution.

The DNA samples were sent in batches to the Génome Québec Innovation Centre, CWRU Genomics Core, and Novogene Corporation for sequencing, as described in the previous studies (Kim and Cullis, 2017; Li and Cullis, 2021). The Illumina HiSeq^®^ 2000 PE100 and HiSeq^®^ 2500 PE150 platforms were used to generate 10,358,444 to 358,941,018 reads equivalent to 1.0 Gb to 35.9 Gb of raw data for the 84 samples. An average of 10.7% of the reads were aligned to the chloroplast genome with a coverage of 8177×. All raw reads were uploaded to the NCBI SRA database (https://www.ncbi.nlm.nih.gov/sra) under accession number PRJNA779273.

### Chloroplast genome assembly and annotation

The reference chloroplast genome of *T. esculentum* was assembled *de novo* previously using a hybrid method (Kim and Cullis, 2017). ABySS (Simpson et al., 2009; https://www.bcgsc.ca/resources/software/abyss) was first used to get contigs from the NGS short reads, then the contigs were mapped to the 3GS PacBio long reads by DBG2OLC (Ye et al., 2016; https://github.com/yechengxi/DBG2OLC) and further assembled according to sequence overlaps. The chloroplast reference genome was re-annotated in this study using BLAST, Expasy (Gasteiger, 2003; https://web.expasy.org/translate), and CPGAVAS2 (Shi et al., 2019; http://47.96.249.172:16019/analyzer/annotate), and the results were uploaded to NCBI GenBank with accession ID KX792933.1.

### Comparative analysis of chloroplast genomes

The paired-end Illumina reads of the 84 individuals were aligned to the *T. esculentum* chloroplast reference genome using Bowtie 2.2.1 (Langmead and Salzberg, 2012) on CyVerse Discovery Environment platform (https://de.cyverse.org). SAM_to_Sorted_BAM-0.1.19 was used for format changes and indexing the BAM files. SNP and indels were obtained using Calling SNPs INDELs with SAMtools BCFtools and BAM-to-SHOREmap3.8, available on CyVerse. The variation data (including reference/variant alleles and corresponding genomic positions and frequencies) from all generated VCF files were transferred into the same spreadsheet. The identified variations were further verified by visualizing the alignments in IGV. Only variants which had Phred scores above 20 and were present in strands in both directions were retained to avoid interference from low-quality reads and strand bias. Low-frequency heteroplasmic polymorphisms were manually identified in IGV with a cutoff frequency of 2%.

The complete chloroplast genomes of the 84 marama individuals were obtained by correcting the reference cp genome according to the alignment result with the help of contigs from directly assembling the Illumina reads using ABySS.

A sliding window analysis was performed in DnaSP6 (Rozas et al., 2017; http://www.ub.edu/dnasp), with a window length of 1200 bp and a step size of 400 bp, to calculate the nucleotide diversity (Pi) of the cp genomes from the 43 independent marama individuals collected in various geographic regions (**Table S1**).

Intraspecific and interspecific divergent regions in the chloroplast genome were detected by mVISTA (Frazer et al., 2004; https://genome.lbl.gov/vista/mvista/about.shtml) using the Shuffle-LAGAN pairwise alignment algorithm. The chloroplast genomes of four selected marama samples and the related species *T. fassoglense* (NC_037767.1) were used as the input with the *T. esculentum* cp genome (KX792933.1) as the reference.

### Simple sequence repeat analysis and phylogenetic tree construction

The two types of *T. esculentum* chloroplast genomes were used separately to identify microsatellites by MISA (Beier et al., 2017; https://webblast.ipk-gatersleben.de/misa). The minimum number of repetitions was set to 10, 6, 5, 5, 5, 5 for the searches of mononucleotide, dinucleotide, trinucleotide, tetranucleotide, pentanucleotide and hexanucleotide repeats, respectively.

The complete chloroplast genomes of 43 independent marama individuals were aligned with the cp genomes of three outgroup species, *T. fassoglense* (NC 037767.1), *Glycine max* (NC_007942.1), and *Millettia pinnata* (NC_016708.2) by Muscle 3.8.31 (Edgar, 2004; https://www.drive5.com/muscle) with two iterations (maxiters = 2). The result was used to draw a Maximum Likelihood (ML) phylogenetic tree using the Jukes-Cantor model and the Tamura-Nei model with 1000 bootstrap replicates in MEGA 11 (Kumar et al., 2008; https://www.megasoftware.net). The overall topology was validated by the Maximum Parsimony (MP) method using the Subtree Pruning Re-grafting (SPR) algorithm with 1000 bootstrap replicates in MEGA 11.

## Results

### Chloroplast genome characteristics

An average of 10.7% of the WGS reads mapped to the marama reference cp genome in the 84 individuals. The average read depth of the chloroplast DNA was about 8175x (**Table S1**). The estimated chloroplast genome lengths ranged from 161,537 bp to 161,580 bp, mainly due to the different lengths of LSC regions, as the remaining regions were of the same length in the 84 individuals.

Two germplasms, Type 1 and Type 2, were found in the studied marama plants with distinct chloroplast genomes. All marama plants collected from UP Farm and UNAM Farm had the exactly the same chloroplast genome (Type 2), which was different from the previously assembled marama reference chloroplast genome (KX792933.1) at 122 loci and at a 230 bp inversion. The chloroplast genomes of the remaining marama samples collected from different geographic locations in Namibia were similar but not completely identical to the reference, termed Type 1.

The LSC region of Type 2 cpDNA is 25 bp longer than the Type 1 reference cp genome built up on a Namibian individual in the previous study (**Table 1**) (Kim and Cullis, 2017). There was no difference in the number of genes contained in the cpDNA of the 84 individuals.

**Table 1:** Comparison of genomic features of the two types *T. esculentum* chloroplasts.

	Type 1 (KX792933.1)	Type 2 (OP271860)
Size (bp)	161,537	161,562
LSC (bp)	86,113	86,138
SSC (bp)	13,632	13,632
IR (bp)	30,896	30,896
Total genes	129 (18)	129 (18)
Protein coding genes	84 (7)	84 (7)
tRNA genes	37 (7)	37 (7)
rRNA genes	8 (4)	8 (4)
GC content (%)	36.03	36.03

The numbers in parentheses indicate the number of genes with two copies in the *T. esculentum* chloroplast genome. The chloroplast genomes of two individuals are compared here, with annotation deposited in GenBank under accession numbers KX792933.1 and OP271860, respectively.

The annotation of *T. esculentum* cpDNA has been done previously but has required corrections (Kim and Cullis, 2017). The boundaries of some previously annotated genes and newly identified tRNA genes were updated (**Tables S2, 3**). Two copies of *ycf15* should be pseudogenes since they lack valid start codons. The genes *psbL* and *ndhD* have an ACG start codon, and plant plastids have been reported to have a C~U editing mechanism to convert ACG to AUG (Hoch et al., 1991). There are two copies of *rps19*, the one on IRb is a pseudogene due to lack of complete 3’ end sequence, and the other copy, starting with an alternative atypical GTG start codon on IRa and ending on LSC, is complete and functional. The chloroplast genes *rps19*, *psbC, ycf15*, and *infA* possess a GTG initiation codon, which was also reported before (Hirose et al., 1999).

### Chloroplast genome variant analysis

**Figure 2:**
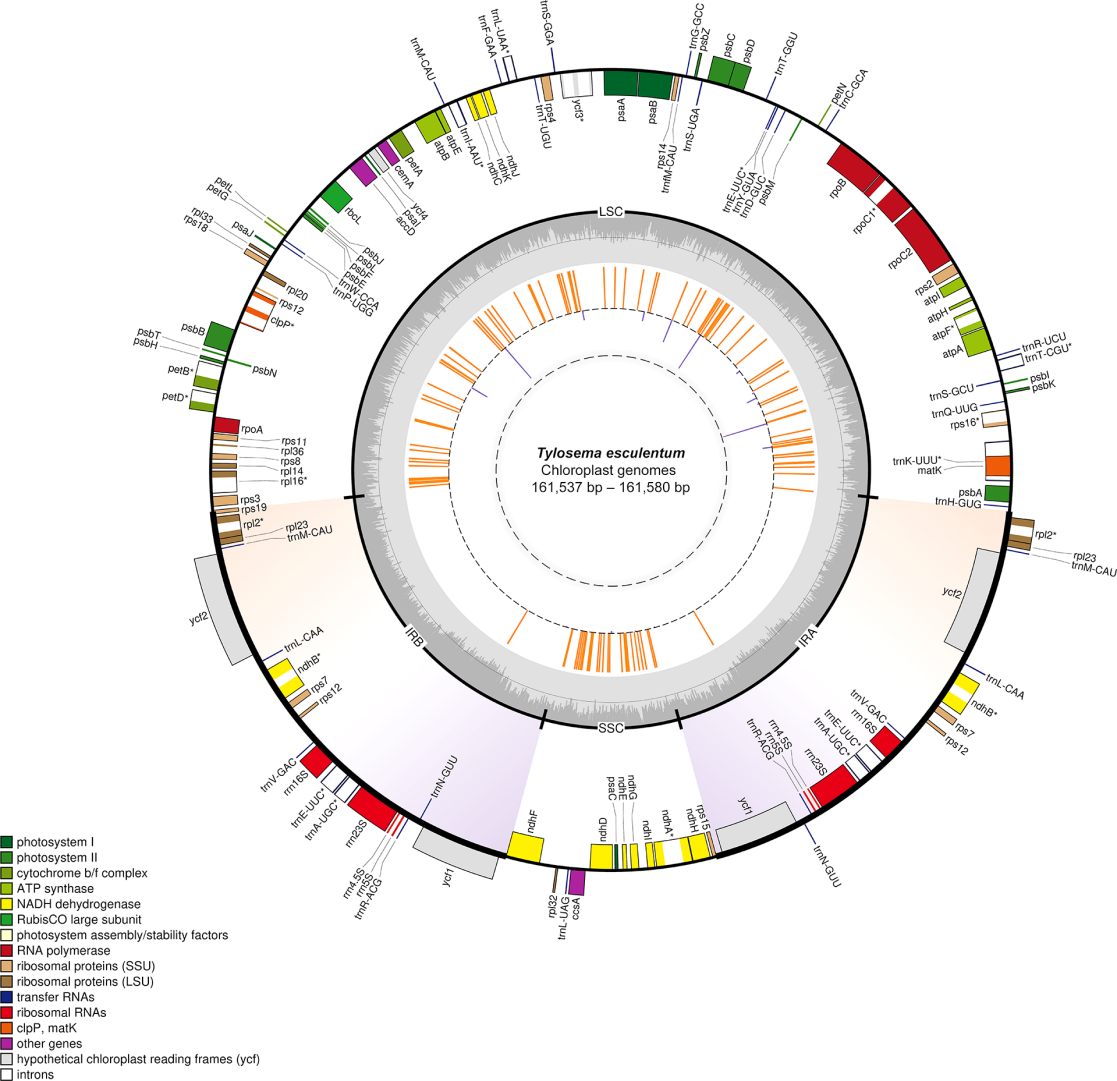
Circular gene map of the plastid genomes of *T. esculentum* drawn by OGDRAW. Genes inside the circle are transcribed clockwise, while genes outside the circle are transcribed counter clockwise. Genes are colored according to their function. Genes with introns are marked with an asterisk. GC content is indicated by the dark grey shading of the inner circle. The two inverted repeats are highlighted by gradient colors. The loci where Type 2 plants and Type 1 plants differ are represented by the orange lines in the inner circle and loci with differences in Type 1 plants from different geographical regions are represented by the purple lines. The length of the lines reflects the number of individuals with the alternative alleles.

As can be seen from the distribution of variation shown in the inner circle in **Figure 2**, numerous variations are located in the LSC and SSC regions. However, the region where the two large inverted repeats are located has barely no variation except that each contains a locus with three consecutive base substitutions. All orange line segments have the same length, indicating that all Type 2 plants are different from the reference cp genome at these loci.

**Figure 3:**
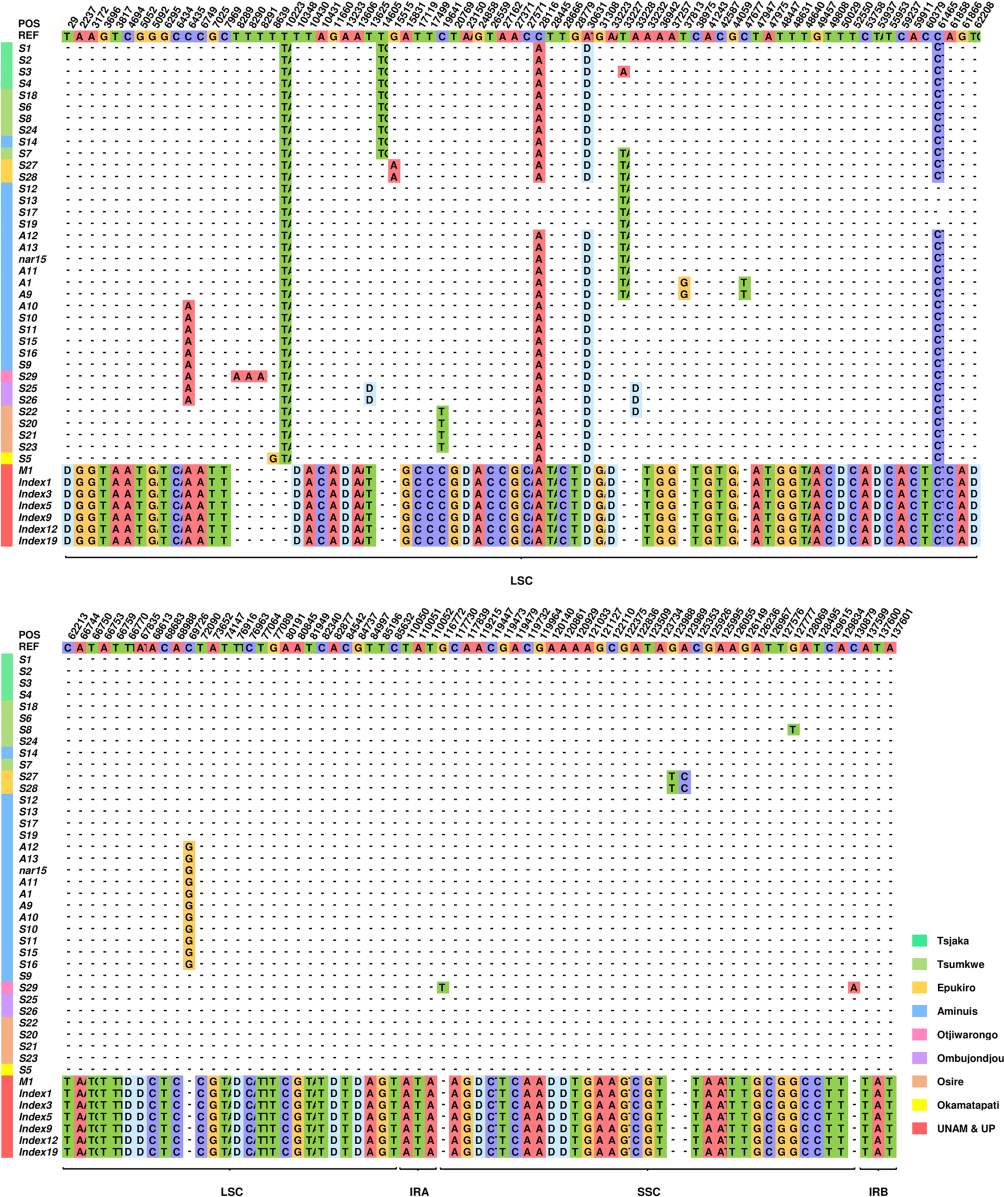
Distribution map of all variations in 43 independent *T. esculentum* individuals. This figure includes only loci that differ in these plants, and the top row shows the sequence of the reference genome. Only bases different from the reference are exhibited, others are represented by dashes. To save space, only the first two bases of insertions are displayed. The letter D means deletions. The color bar to the left of the plant ID shows the source of the sample.

As shown in **Figure 3**, a total of 147 variants were distributed among 15 haplotypes (**Table S4**). Based on these variations, the chloroplast genomes of these plants can be clearly divided into two categories. The two types differ from each other at 122 loci (**Table 2**). No differences were found between the 7 Type 2 plants, but the 36 Type 1 individuals could then be divided into subgroups based on the other 21 loci. Individuals from the same geographic location are likely to have the same cpDNA. Some substitutions appeared only in plants from the same geographic location.

**Table 2:** Total counts of variation in the chloroplast genomes by Calling SNPs with Samtools when aligning the cpDNA of Type 1 and Type 2 plants to the previously published marama reference chloroplast genome (KX792933.1).

Variation Type	Type 2 vs. Ref.	Type 1 vs. Ref.
Deletion	17	3
Insertion	19	5
SNP	90	17
Total	126 (122exclusive)	25

There were 105 SNPs in total, accounting for 71.4% of all variations. 31 SNPs but no indels were found in the coding sequence, 17 of which were silent mutations and 14 were nonsynonymous. Variations that altered the resulting amino acid sequence accounted for 45.2% of the total variation found in coding sequences and 9.5% of all variation in the cpDNA of the 84 individuals. The specific positions and effects of these SNPs are shown in **Table 3**. Genes including *rpoC2*, *rpoB*, *rpoA*, *ndhF*, *ndhD*, *ndhH*, *rps3* contain multiple SNPs in CDS. The introns of genes *ndhA*, *petB* and *trnK* have three or more variation sites (**Table 4**).

**Table 3:** Variation positions found in the marama chloroplast coding sequence and their effect on the resulting amino acid sequence.

Gene Abbreviation	Position	Reference	Mutation	Amino acid change
*matK*	3172	A	G	synonymous
*atpA*	11660	G	A	synonymous
*atpI*	15515	G	A	P58S
*rpoC2*	17119	T	C	synonymous
*rpoC2*	17499	T	C	S1177G
*rpoC2*	19641	C	T	G463S
*rpoC2*	20769	T	G	N87H
*rpoB*	24858	G	A	synonymous
*rpoB*	26559	T	C	synonymous
*rpoB*	27162	A	C	I48M
*psaB*	41243	A	G	synonymous
*psaA*	42587	C	T	synonymous
*ndhC*	52550	T	C	T24A
*atpB*	55953	T	C	synonymous
*accD*	60379	C	T	synonymous
*rps18*	69726	C	G	T117S
*rpoA*	80191	A	C	N264K
*rpoA*	80845	A	G	synonymous
*rps3*	85196	T	G	synonymous
*rps3*	85632	C	T	synonymous
*ndhF*	117730	C	A	A495S
*ndhF*	117839	A	G	synonymous
*ccsA*	122110	C	A	L294I
*ndhD*	122836	A	C	S251A
*ndhD*	123509	T	G	L26F
*ndhD*	123734	A	T	synonymous
*ndhG*	125353	C	T	synonymous
*ndhI*	126236	A	G	synonymous
*ndhA*	126967	T	C	synonymous
*ndhH*	129615	C	T	R230H
*ndhH*	129834	A	T	F157Y

For nonsynonymous substitutions, for example, P58S indicates a change of the 58^th^ amino acid from Proline to Serine.

**Table 4:** Number of variations found in introns of *T. esculentum* chloroplast genes.

Gene	Product	SNP CTS	Indel CTS	Indel Type
*ndhA*	NADH-plastoquinone oxidoreductase subunit 1	4		
*rps16*	Ribosomal protein S16	2	1	1 bp INS
*atpF*	ATP synthase subunit b		1	1 bp DEL
*rpoC1*	RNA polymerase beta subunit		1	5 bp DEL
*clpP*	ATP-dependent Clp protease proteolytic subunit	2		
*petB*	Cytochrome b6	1	3	5 bp DEL/1 bp INS/10 bp INS
*rpl16*	ribosomal protein L16	1	1	1 bp DEL
*trnL*	tRNA-Leu	1		
*trnK*	tRNA-Lys	3		
*trnV*	tRNA-Val	1		

CTS, Counts.

### Identification of variable regions

**Figure 4:**
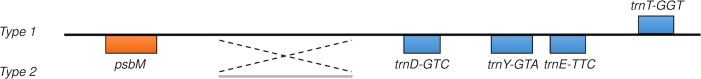
Schematic diagram of the 230 bp inversion in the *psbM-trnD* intergenic spacer. A 230 bp sequence (30,663-31,480 in the marama reference cpDNA) is reversed in all Type 2 individuals. However, all Type 1 plants are consistent with the reference genome in this area. The surrounding region contains a set of closely located tRNA genes.

The average nucleotide diversity per site is 0.00032. However, a high π value (0.02070 in a 1200 bp window) was detected in the region where the *psbM-trnD* intergenic spacer is located. A 230 bp inversion was found between *psbM* and 4 closely located tRNA genes (**Figures 4**, **S1**). Sequence alignment indicated that all Type 1 plants were identical to the reference genome in this region, but this 230 bp sequence was inverted in all Type 2 samples (**Figure S3**).

**Figure 5:**
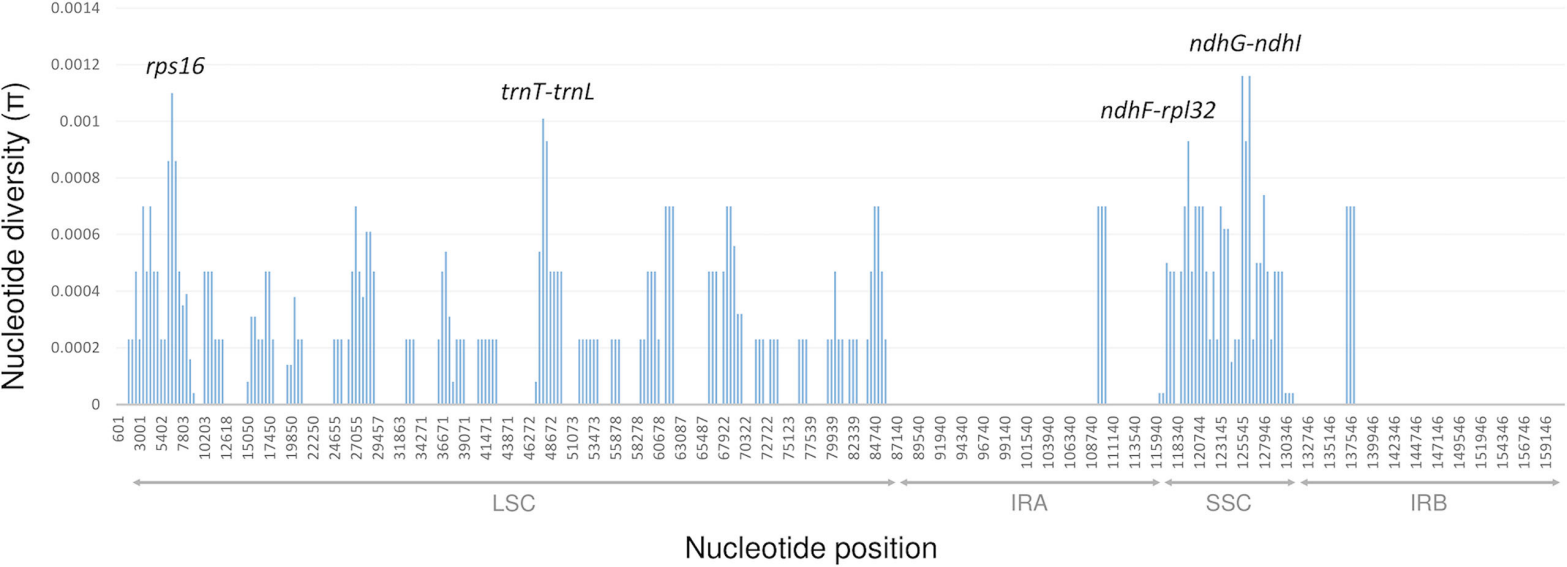
Sliding window analysis of the chloroplast genomes of the 43 independent *T. esculentum* individuals (window length: 1200 bp, step size: 400 bp). The *x*-axis shows the midpoint position of each window. 30,949-31,218, where the 230 bp inversion is located was excluded from this analysis (**Figures S1, 2**).

Nucleotide diversity within each 1200 bp window of the rest of the chloroplast genome ranged from 0 to 0.00116 (**Figure 5**). The most variable regions included *trnT-trnL*, *ndhG-ndhI* intergenic spacers, and the intron of *rps16* (all with a π value above 0.001). Although the marama cp genome was diverged into two clades, and multiple subgroups exist within them, overall, the cpDNA of marama remains highly conserved.

**Figure 6:**
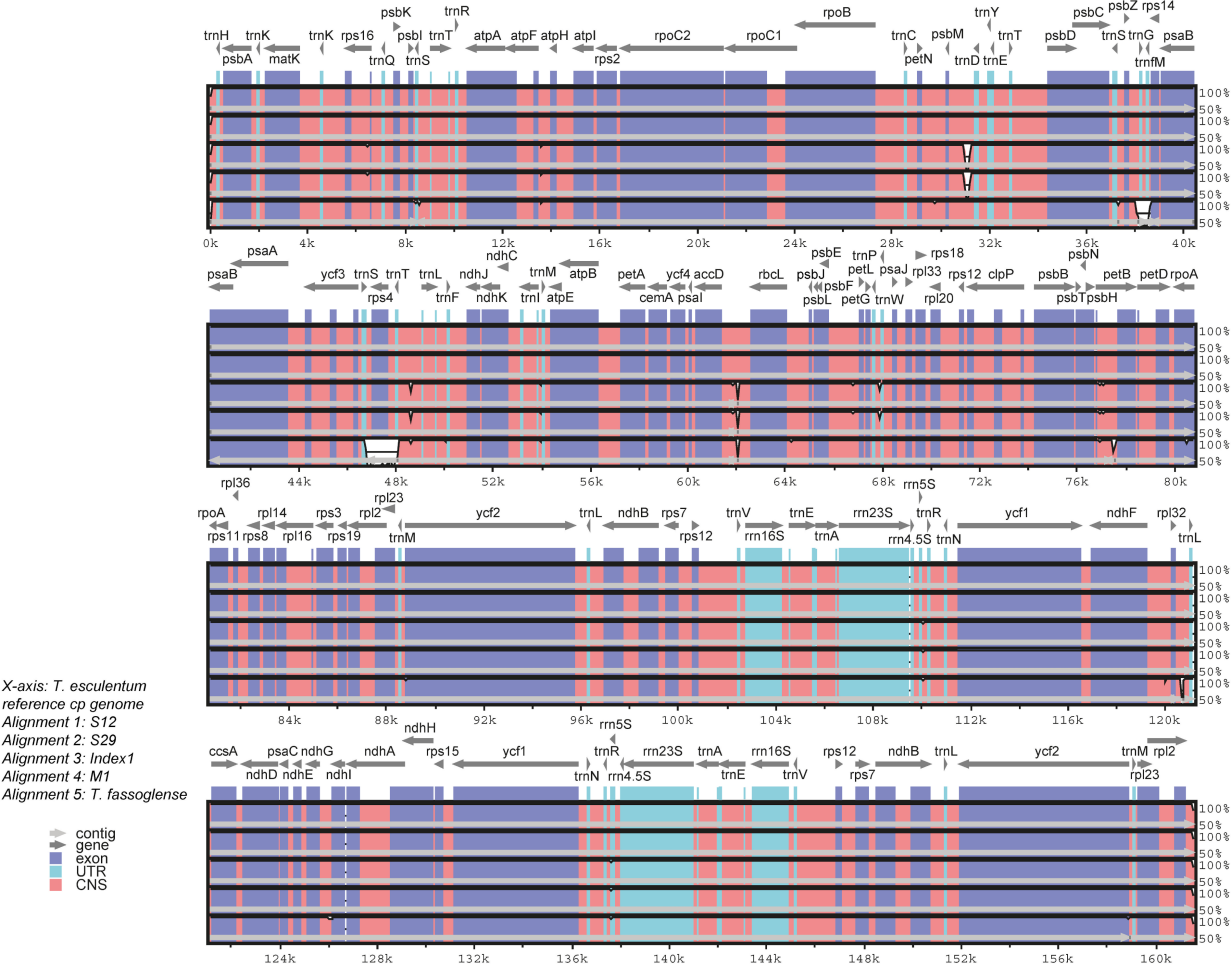
Comparison of cpDNA from four *T. esculentum* individuals (Type 1: S12 and S29, and Type 2: Index1 and M1) and the related species *T. fassoglense* (NC_037767.1) by mVISTA Shuffle-LAGAN alignment. The plastome of *T. esculentum* (KX792933.1) was used as the reference. White blanks indicate regions of sequence divergence. The *x*-axis represents the position in the chloroplast DNA. The *y*-axis shows the similarity of sequence alignment, ranging from 50% to 100%. The location and transcription direction of the cp gene are labeled at the top of the block. Conserved exons, introns and noncoding regions are marked on the graph with different colors.

Pairwise comparisons of cpDNA of Type 1 and Type 2 marama samples and the related species *T. fassoglense* by mVISTA alignment revealed low levels of sequence divergences between the chloroplast genomes (**Figure 6**). In the coding sequence of *rps4* and the positions of the genes *trnG* and *trnM*, *T. fassoglense* showed clear differences from *T. esculentum*. Furthermore, at the 5’ end of *accD*, the two Type 2 plants differed from the Type 1 plants but were more consistent with *T. fassoglense*. Type 2 plants and Type 1 plants also diverged between *psbM* and *trnD*, due to the presence of a 230 bp inversion. Another divergence was found at 48,640, where the Type 2 plants contained a 48 bp insertion that made the sequence “taattagaattaagtaattataaa” triplicated in this region and shown as a tandem repeat.

**Figure 7:**
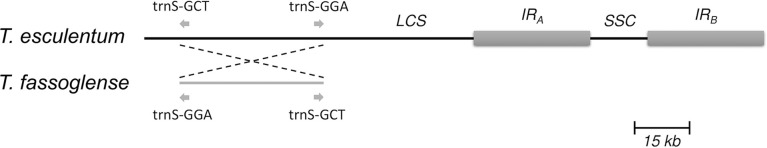
Diagram showing a 38,314 bp inversion between the two *trnS* genes in the LSC region of *T. esculentum* and *T. fassoglense* cpDNA. This was also reported by Wang et al. (2018).

Blastn alignment of the cpDNAs of *T. esculentum* and *T. fassoglense* revealed a 38,314 bp long inversion in the LSC region (marama reference cpDNA: 8,427 to 46,740) (**Figures 7**, **S4**), which was also reported before by Wang et al. (2018). This inversion accounted for 44.5% of the length of the LSC region and 23.7% of the total length of the cpDNA, containing 17 protein-coding genes and 12 tRNA genes. Both boundaries are the 3’ ends of the two *trnS*. The sequences of the two *trnS* genes share 78% (69/88) similarity. Therefore, it was speculated that the recombination that occurred at the two *trnS* genes reversed the intermediate sequence.

### Phylogenetic construction

**Figure 8:**
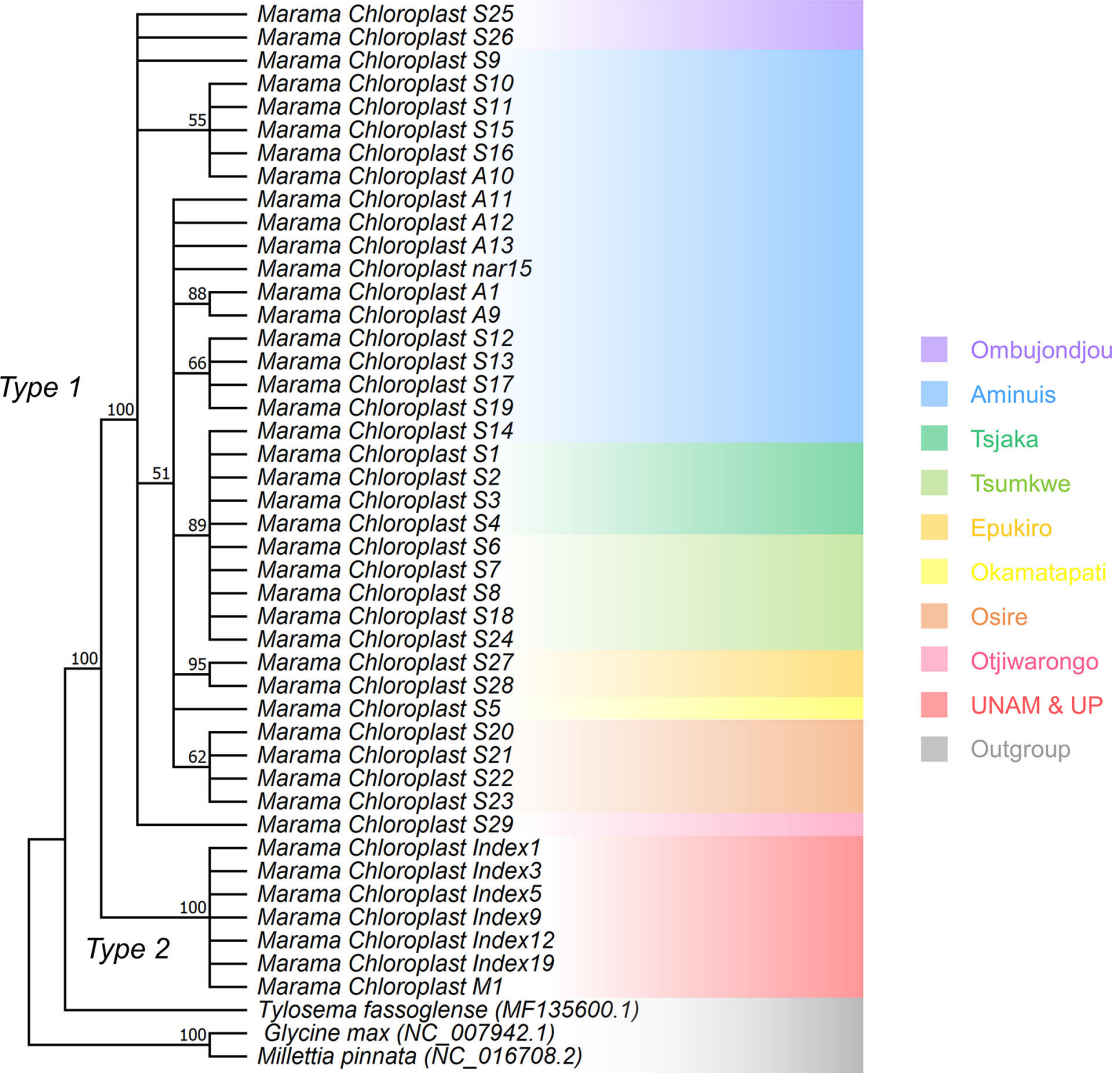
Maximum Likelihood (ML) phylogenetic tree based on the Jukes-Cantor model and the Tamura-Nei model showing the relationship of the chloroplast genomes of 43 independent *T. esculentum* individuals and three other Fabaceae species. *T. fassoglense* (MF135600.1), *Glycine max* (NC_007942.1), and *Millettia pinnata* (NC_016708.2) were used as outgroups. Bootstrap values from 1000 replicates were marked on the branches with 50% as cutoff. This was validated by the Maximum Parsimony (MP) method using the Subtree Pruning Re-grafting (SPR) algorithm with 1000 bootstrap replicates in Mega 11. Background colors indicate the geographic origin of the samples.

The phylogenetic study indicated that the cpDNA of the marama samples were clearly divided into two clades (**Figure 8**). The cpDNAs of the Type 2 samples were basically the same and very conserved, but Type 1 samples could be further divided into multiple subgroups. The differences found between Type 1 plants were potentially related to the geographical origin of the samples, however, this needs to be proved by experiments with larger sample sizes. Diverged cpDNAs also appeared in plants from the same geographic location. For example, at least four cpDNA subgroups were found in the marama plants from Aminuis. One individual of them, S14, was unexpectedly found to be highly similar to the samples collected in the geographically distant Tsumkwe region. More samples collected in these two regions are needed for comparison.

### SSRs and heteroplasmy analysis

**Figure 9:**
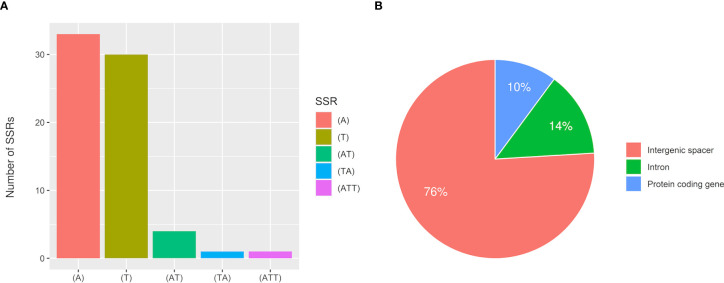
Statistical analysis of SSRs in *T. esculentum* chloroplast genome by MISA. **(A)** Number of identified SSR motifs in different repeat types **(B)** Location distribution of SSR repeats.

MISA analysis found a total of 79 SSRs, most of which were A or T mononucleotide repeats accounting for 79.7% (**Figure 9**). There were 5 AT or TA dinucleotide repeats, only one trinucleotide repeat ATT, and 10 compound SSRs. 60 SSRs were in noncoding intergenic regions, 11 were in introns, and 8 were in the coding sequences of genes including *rpoC2*, *rpoB*, *atpB*, *accD*, *rps18*, *clpP*, *ndhF*, and *rps19*. The analytical results for Type 1 samples and Type 2 samples were consistent.

**Figure 10:**
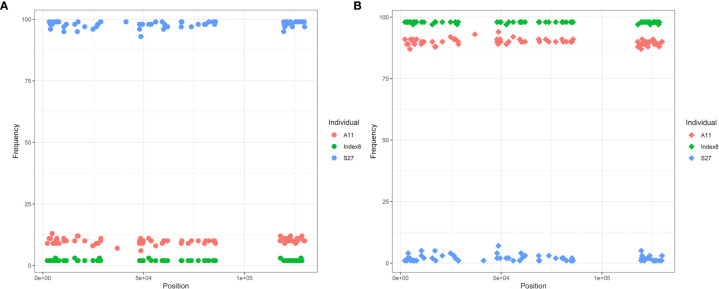
Diagram of allele frequencies of the 105 SNP loci different in Type 1 and Type 2 plants. **(A)** Frequency of the Type 2 alleles at these 105 loci. **(B)** Frequency of the Type 1 alleles at these loci. Only the frequencies of SNPs were recorded and compared here, as accurate indel frequencies were hard to obtain.

By examining allele frequencies in IGV, we found that three individuals contained both Type 1 and Type 2 cpDNA (**Figure 10**). Index8, as a Type 1 individual, also contained the Type 2 alleles among the 122 inter-type difference loci, the frequency of which remained consistent around 2%, and vice versa for S27. The minor alleles are easily ignored because their frequencies are well below the default cutoff for Calling SNPs with Samtools, and they are also difficult to distinguish from sequencing and alignment errors. With one exception, as a Type 1 individual from Aminuis, A11 also contained the Type 2 alleles in its cp genome with a frequency of approximately 11% among these 122 loci. The read depth of these minor alleles is about 450x, much higher than the nuclear DNA coverage (35-50x) indicating the existence of heteroplamsy in the marama chloroplast genome. In the cp genomes of other individuals, heterogeneity only existed at some loci but not others. If the cutoff value is further reduced from 2% to lower, it is expected to see more heteroplasmy. It is speculated that paternal leakage resulted in the presence of both types of chloroplast DNAs in the same individual. The frequency of the minor alleles is too low to be detected effectively, and it is uncertain whether there is selection in favor of certain gene alleles or DNA segments over others.

## Discussion

As an orphan species, marama can not only survive in extreme environments of Africa, but it also provides edible and nutritious seeds, making the domestication and genetic research of the bean very valuable. Molecular studies of the marama chloroplast genome can help us better understand this species from an evolutionary perspective, as well as identify existing polymorphism that could be associated with phenotypes of interest, which will aid marama domestication and breeding.

By conducting DNA sequencing on a large number of individuals, this study aimed to compare the chloroplast genomes of marama plants at the individual and population level, and to investigate intraspecific variations that exist among them. A total of 84 samples (43 independent individuals) were collected from different geographic locations in Namibia and Pretoria. The cp genomes of these plants were compared to identify polymorphisms, including SNPs, indels and genomic structural variations. Highly variable cp genes and genomic regions were discussed. Phylogenetic analysis indicated the existence of two distinct germplasms in marama, with chloroplast genomes distinguished from each other at 122 loci. There was also a structural difference, a 230 bp inversion, found between the two types of chloroplast genomes, which provides valuable information for studying germplasm evolution (Palmer et al., 2000). Our study also confirmed a previous finding by Wang et al., in 2018 that a large inversion of 38,314 bp exists between the two *trnS* genes in the LSC region of *T. esculentum* and *T. fassoglense* plastomes. Large inversions of cpDNA within the same genus are uncommon, although they have been reported previously in genera such as *Artemisia* and *Astragalus* (Johansson, 1999; Charboneau et al., 2021). This large inversion is not fully reflected in our unscaled tree (**Figure 8**). In the scaled tree, long branch lengths were expected to be seen between the two species, however, all marama individuals of the same type would be clustered together, thus obscuring intra-type differences. One type of marama cpDNA was found to be relatively conserved with very low diversity. The other type appeared to have more variability within the major genome present, with a total of 25 intra-type variations and these variations seemed to be distributed within specific geographic regions.

Of all the variants detected, 31 SNPs were located in the coding sequence of marama cpDNA, 14 of which were nonsynonymous, altering the synthesized protein sequence. Both *rpo* genes and *ndh* genes appeared to be less conserved. The coding sequences of the genes *rpoC2* and *rpoB*, the introns of the genes *ndhA*, *petB*, *trnK*, and *rps16*, and the intergenic spacers *trnT-trnL*, *ndhG-ndhI* were found to be more variable compared to the other regions of the marama plastome. Whether these genomic variations and the formation of distinct chloroplast genomes have anything to do with the adaptation of marama to harsh environments will be an interesting question to explore. Understanding how these polymorphisms are associated with any particular phenotype, like plant stress responses, still requires further large-sample statistical analysis to link phenotypic data to the different genotypes (Members of the Complex Trait Consortium, 2003). Therefore, SSRs and long sequence repeats were also analyzed, which could be used to establish molecular markers to aid in rapid genotyping of large numbers of samples.

In addition, it was interesting to find heteroplasmy in the cpDNA of some marama individuals. Both types of cpDNA co-existed in those individuals, but one predominated and the other had a very low frequency between 0% and 2%. However, there was one exception. In one individual, the minor cpDNA frequency reached 11%. Previous studies speculated that the occasional leakage of paternal cytoplasmic information led to heteroplasmy (McCauley et al., 2005), but the effect of the long-term accumulation of spontaneous *de novo* mutations in cpDNA could not be ruled out. Why plant cells can possess both organelle genomes at the same time and maintain the ratio of the two to a certain extent, and whether this ratio changes during cell development as described by the animal mitochondrial bottleneck hypothesis remains unknown (Cao et al., 2007). Understanding the genetic mechanisms behind this could help us better understand the function and inheritance of the organelles.

In general, this in-depth study of the chloroplast genome of marama not only contributes to marama variety identification, but it also helps us better understand the origin and evolution of the bean. All these are conducive to the domestication and breeding of marama in the future.

## Data availability statement

The data presented in the study are deposited in GenBank (https://www.ncbi.nlm.nih.gov/genbank) with accession numbers KX792933.1 and OP2371860, and in the Sequence Read Archive (SRA) (https://www.ncbi.nlm.nih.gov/sra), accession number PRJNA779273.

## Author contributions

JL carried out the bioinformatics assembly and data analysis and drafted the manuscript. CC provided extracted DNAs, was involved in assembly of chloroplast genome, and assisted in writing and editing the manuscript. All authors contributed to the article and approved the submitted version.
