# Supplementary material for: Comparative analysis of 84 chloroplast genomes of Tylosema esculentum reveals two distinct cytotypes
Source: Front Plant Sci. 2023 Jan 31;13:1025408. doi: 10.3389/fpls.2022.1025408 (PMC9927231; doi:10.3389/fpls.2022.1025408)
Supplement: Supplementary file 1 [file DataSheet_1.pdf]

Supplementary Materials

Table S1. Information on the source and sequencing details of the 84 samples

Sample	Plant source	Raw reads	Raw data	CP align rate (%)	CP length (bp)
M17	UNAM Farm Progenies	96750044	14512506600	8.37	161562
S_35	UNAM Farm Progenies	86626894	12994034100	19.2	161562
S_19	UNAM Farm Progenies	55375806	8306370900	14.81	161562
S_4	UNAM Farm Progenies	73435564	11015334600	12.32	161562
S_13	UNAM Farm Progenies	217730028	32659504200	14.62	161562
S_27*	UNAM Farm Progenies	107264134	16089620100	0.29	161562
S_20	UNAM Farm Progenies	55371622	8305743300	9.15	161562
S_30	UNAM Farm Progenies	106501502	15975225300	17.64	161562
S_33	UNAM Farm Progenies	130811500	19621725000	17.54	161562
M7	UNAM Farm Progenies	172383630	25857544500	17.78	161562
M8	UNAM Farm Progenies	123934796	18590219400	17.54	161562
M1 #	UNAM Farm Progenies	170816890	25622533500	12.84	161562
M2	UNAM Farm Progenies	194335620	29150343000		161562
M11	UNAM Farm Progenies	125005082	18750762300	14.32	161562
M12	UNAM Farm Progenies	128360580	19254087000		161562
M15	UNAM Farm Progenies	134439570	20165935500	15.99	161562
M16	UNAM Farm Progenies	132448260	19867239000	18.9	161562
M23	UNAM Farm Progenies	134475978	20171396700	15.07	161562
M22	UNAM Farm Progenies	111447592	16717138800	16.11	161562
M24	UNAM Farm Progenies	127392478	19108871700	19.48	161562
M26	UNAM Farm Progenies	225925198	33888779700	1.18	161562
M28	UNAM Farm Progenies	193804308	29070646200	11.85	161562
M25	UNAM Farm Progenies	145937916	21890687400	13.13	161562
N29	UNAM Farm Progenies	147710832	22156624800	17.32	161562
M31	UNAM Farm Progenies	177899436	26684915400	10.75	161562
M34	UNAM Farm Progenies	124221640	18633246000	6	161562
M36	UNAM Farm Progenies	125524008	18828601200	17.31	161562
M37	UNAM Farm Progenies	185151336	27772700400	15.11	161562
M38	UNAM Farm Progenies	168052326	25207848900	13.35	161562
M40	Namibia Unknown	135702768	20355415200	18.56	161562
Index1 #	UP Farm	41499124	4149912400	7.25	161562
Index10	UP Farm Progenies	36382172	3638217200	12.1	161562
Index11	UP Farm Progenies	34631932	3463193200	11.62	161562
Index12 #	UP Farm	39339004	3933900400	13.83	161562
Index19 #	UP Farm	35994474	3599447400	17.91	161562
Index3 #	UP Farm	35991990	3599199000	16.5	161562
Index5 #	UP Farm	34349602	3434960200	38.46	161562
Index8	Namibia Unknown	42747718	4274771800	6.73	161562
Index9 #	UP Farm	34312880	3431288000	13	161562

R1R2	Unknown	358941018	35894101800	6.25	
A1 #	Aminuis Progenies	93324222	13998633300	8.31	161554
A2	Aminuis Progenies	93445492	14016823800	6.2	161554
A3	Aminuis Progenies	84081976	12612296400	11.28	161554
A4	Aminuis Progenies	60965538	9144830700	13.29	161554
A5	Aminuis Progenies	91044746	13656711900	13.57	161554
A6	Aminuis Progenies	89785050	13467757500	17.46	161554
A7	Aminuis Progenies	86000118	12900017700	14.26	161554
A8	Aminuis Progenies	57087632	8563144800	12.53	161554
A9 #	Aminuis	62849376	9427406400	13.37	161554
A10 #	Aminuis	75633554	11345033100	19.93	161553
A11 #	Aminuis	92851730	13927759500	4.74	161554
A12 #	Aminuis	84374620	12656193000	10.76	161554
A13 #	Aminuis	83450100	12517515000	4.43	161554
nar15 #	Aminuis	81618952	12242842800	14.2	161554
nar16	UP Farm Progenies	96595344	14489301600	10.25	161562
S1* #	Tsjaka	10358444	1035844400	0.29	161579
S2 #	Tsjaka	20343934	2034393400	3.82	161579
S3 #	Tsjaka	33100100	3310010000	5.37	161579
S4 #	Tsjaka	25428338	2542833800	4.36	161579
S5 #	Okamatapati	27183834	2718383400	6.6	161553
S6 #	Tsumkwe	24185808	2418580800	7.95	161579
S7* #	Tsumkwe	22075112	2207511200	1.79	161580
S8* #	Tsumkwe	16337544	1633754400	3.23	161579
S9 #	Aminuis	25274640	2527464000	5.24	161553
S10 #	Aminuis	28329944	2832994400	7.21	161553
S11 #	Aminuis	26741342	2674134200	7.52	161553
S12 #	Aminuis	29520092	2952009200	5.73	161552
S13 #	Aminuis	NA	NA	NA	161552
S14 #	Aminuis	22928100	2292810000	3.81	161579
S15 #	Aminuis	22856344	2285634400	7	161553
S16 #	Aminuis	25333752	2533375200	6.33	161553
S17 #	Aminuis	23418786	2341878600	7.07	161552
S18 #	Tsumkwe	25764102	2576410200	4.14	161579
S19 #	Aminuis	24407080	2440708000	6.92	161552
S20 #	Osire	25692398	2569239800	5.48	161553
S21 #	Osire	25940358	2594035800	8.04	161552
S22 #	Osire	32107310	3210731000	8.01	161552
S23 #	Osire	35844166	3584416600	7.09	161553
S24 #	Tsumkwe	31973008	3197300800	6.58	161579
S25 #	Ombujondjou	24401422	2440142200	6.91	161551
S26 #	Ombujondjou	32758062	3275806200	5.89	161551
S27 #	Epukiro	28122544	2812254400	7.98	161555

S28 #	Epukiro	31273940	3127394000	7.95	161555
S29 #	Otjiwarongo	25274640	2527464000	5.24	161553

* samples with low chloroplast coverage, **#** 43 independent samples for sequence diversity and phylogenetic analysis

Table S2. Correction of *T. esculentum* chloroplast gene positons

Gene Abbrv.	Former Position	Corrected Position	Region	Function
matK	3812..2215	3612..2269	LSC	Other genes
rps16	5803..5567	6602..6561, 5794..5567	LSC	Self replication
psbK	7559..7753	7568..7753	LSC	Photosynthesis
psbI	8123..8275	8165..8275	LSC	Photosynthesis
atpA	12049..10517	12049..10544	LSC	Photosynthesis
atpF	13437..12117	13437..13294, 12521..12114	LSC	Photosynthesis
rpoC2	19131..16888	21027..16888	LSC	Self replication
rpoC1	24065..21205	24065..23634, 22818..21214	LSC	Self replication
petN	29088..29186	29097..29186	LSC	Photosynthesis
psbC	35402..36862	35441..36862	LSC	Photosynthesis
psaA	43468..41324	43558..41306	LSC	Photosynthesis
ycf3	46381..44327	46396..46256, 45536..45309, 44479..44327	LSC	Unknown
ndhK	52215..51583<br>52377..51592	52215..51556	LSC	Photosynthesis
accD	61305..60364	61401..60349	LSC	Other genes
rbcL	64051..62603	64030..62603	LSC	Photosynthesis
psaJ	68411..68545	68411..68539	LSC	Photosynthesis
rps18	69377..69694	69377..69754	LSC	Self replication
rps12	71291..71163<br>100874..100566	71291..71178, 100808..100566	LSC	Self replication
clpP	72885..71530	73748..73680, 72882..72589, 71748..71482	LSC	Other genes
psbT	75988..76104	75997..76104	LSC	Photosynthesis
psbN	76378..76148	76302..76171	LSC	Photosynthesis
psbH	76398..76628	76407..76628	LSC	Photosynthesis
petB	77650..78336	76758..76763, 77695..78336	LSC	Photosynthesis
petD	79220..79744	78528..78536, 79271..79744	LSC	Photosynthesis
rpoA	80982..79999	80982..79981	LSC	Self replication
rps11	81442..81053	81484..81053	LSC	Self replication
rpl16	83894..83484	84959..84951, 83882..83484	LSC	Self replication
rps3	85802..85146	85802..85137	LSC	Self replication
rpl2	87955..86460	87955..87566, 86894..86460	IRA	Self replication
rpl23	88344..88066	88344..87901	IRA	Self replication
ycf2	90160..95700	88822..95700	IRA	Unknown
ycf15	95879..95968<br>101763..101572	95968..95879 (pseudo)	IRA	Unknown
ndhB	99163..96952	99163..98387, 97707..96952	IRA	Photosynthesis
rrn23S	106037..109461	106651..109462	IRA	Self replication

rpl32	120263..120415	120263..120430	SSC	Self replication
ccsA	121231..122184	121231..122190	SSC	Other genes
ndhD	123955..122441	123937..122441	SSC	Photosynthesis
ndhE	124879..124574	124876..124574	SSC	Photosynthesis
ndhI	126664..126161	126664..126167	SSC	Photosynthesis
ndhA	129120..126745	129120..128584, 127266..126745	SSC	Photosynthesis
rrn23S	141614..138190	141000..138189	IRB	Self replication
rps12	146777..147805	71291..71178, 146843..147085	IRB	Self replication
ndhB	148488..150699	148488..149264, 149944..150699	IRB	Photosynthesis
ycf15	151772..151683	151772..151683 (pseudo)	IRB	Unknown
ycf2	157491..151951	158829..151951	IRB	Unknown
rpl23	159307..159585	159307..159750	IRB	Self replication
rpl2	159696..161191	159696..160085, 160757..161191	IRB	Self replication
rps19	161360..161537	161360..161536 (pseudo)	IRB	Self replication

Table S3. Newly identified tRNA genes in *T. esculentum* chloroplast genome by CPGAVAS2

**Gene Name**	**Position**
trnK-UUU	4583..4547, 1970..1936
trnT-CGU	9043..9077, 9784..9827
trnL-UAA	49078..49112, 49626..49675
trnI-AAU	53845..53815, 53222..53162
trnE-UUC	104535..104566, 105520..105559
trnA-UGC	105624..105660, 106463..106498
trnA-UGC	142027..141991, 141188..141153
trnE-UUC	143116..143085, 142131..142092

Table S4. Population haplotype analysis of the 43 independent marama individuals

**Population**	**n**	**Hn**	**Hd**	**π**
Pretoria	7	1	0	0
Epukiro	2	1	0	0
Aminuis	17	6	0.83088	0.00001
Osire	4	1	0	0
Tsumkwe	5	2	0.4	0
Tsjaka	4	1	0	0
Ombujondjou	2	1	0	0
Okamatapati	1	1	0	0
Otjiwarongo	1	1	0	0

n = number of sequences, Hn = number of haplotypes, Hd = haplotype diversity, π = nucleotide diversity

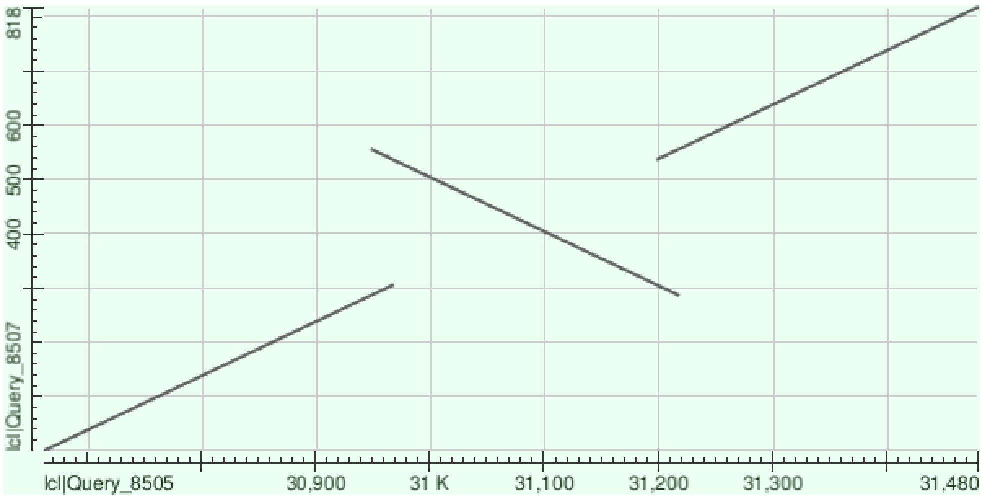

Figure S1. Alignment of cpDNA sequences of Type 1 and Type 2 marama samples revealed the existence of a 230 bp inversion. A portion of the marama reference cpDNA was blasted to the corresponding region of the Type 2 sample and displayed as the dot plot above. The inversion is located at the intergenic region (30,949-31,218) between *psbM* and four closely located tRNA genes, *trnD-GTC*, *trnY-GTA*, *trnE-TTC*, and *trnT-GGT* in the chloroplast genomes of marama.

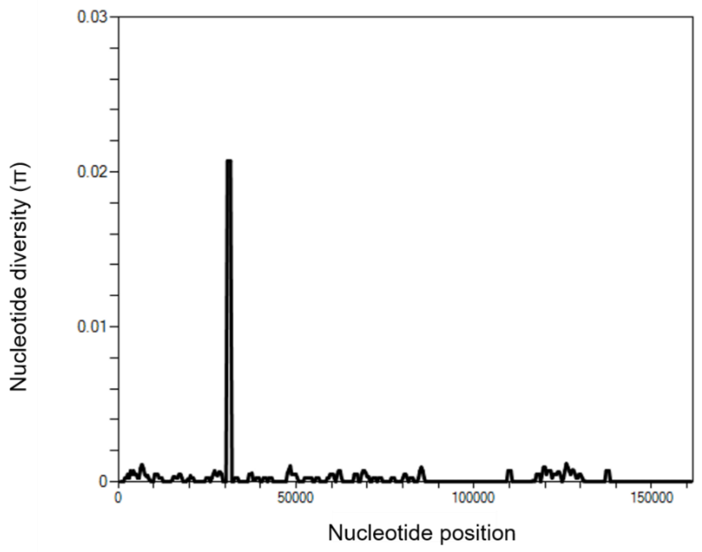

Figure S2. Sliding window analysis of the chloroplast genomes of the 43 independent *T. esculentum* individuals (window length: 1200 bp, step size: 400 bp) The *x*-axis shows the midpoint position of each window.

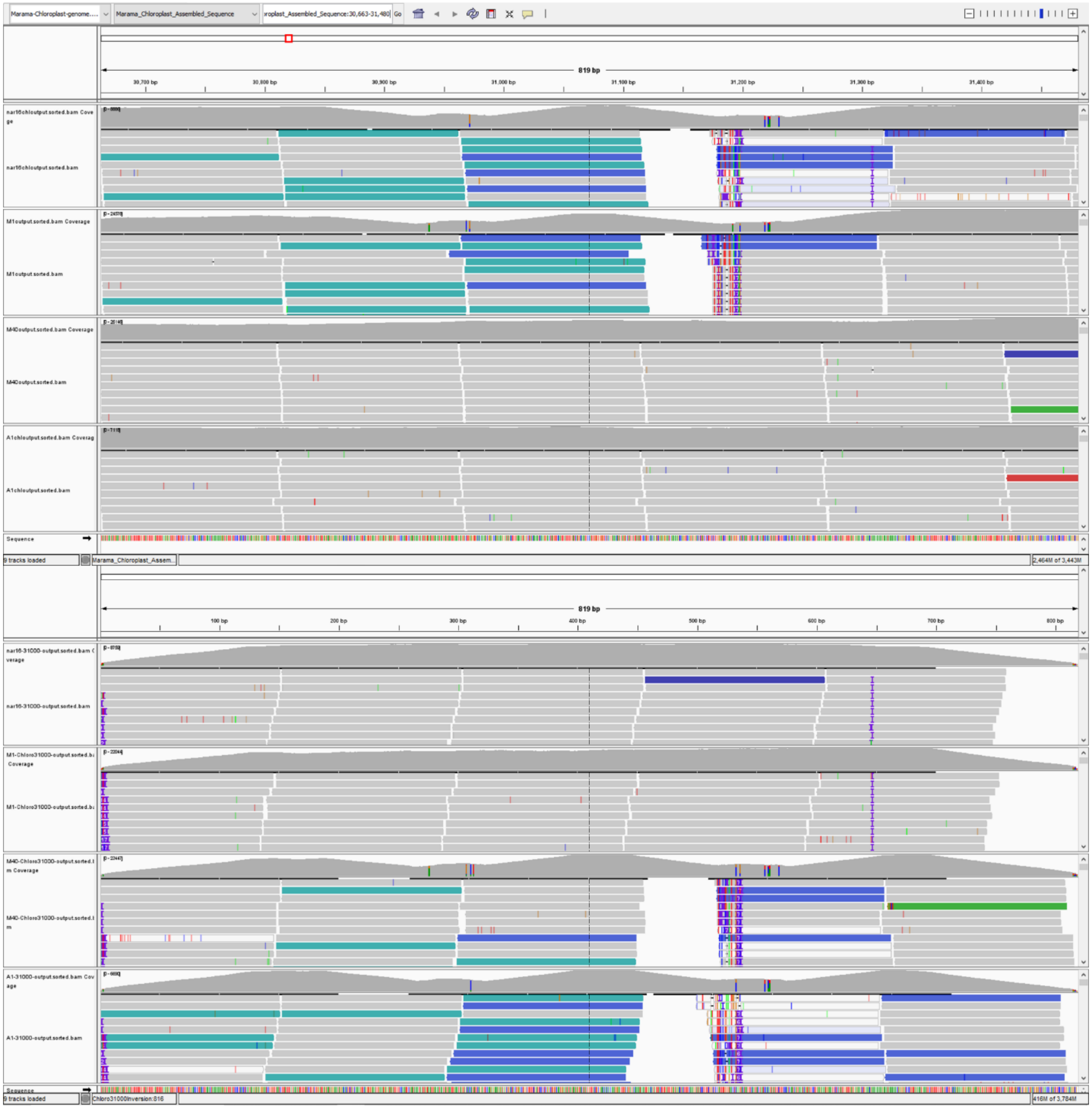

Figure S3. Visualization of sequence alignment in IGV confirmed a 230 bp inversion between 30,949 and 31,218. Top: The Illumina reads from 4 individuals (two Type 2 plants: nar16 and M1, and two Type 1 plants: M40 and A1) were aligned with the reference marama cp genome and distinct gaps could be seen in both Type 2 plants. Bottom: After inverting the 230 bp between 30,949 and 31,218 in the reference cp genome and redid the alignment, the gaps were missing in the two Type 2 samples but appeared in the two Type 1 samples. This was consistent with the rest of Type 1 and Type 2 individuals.

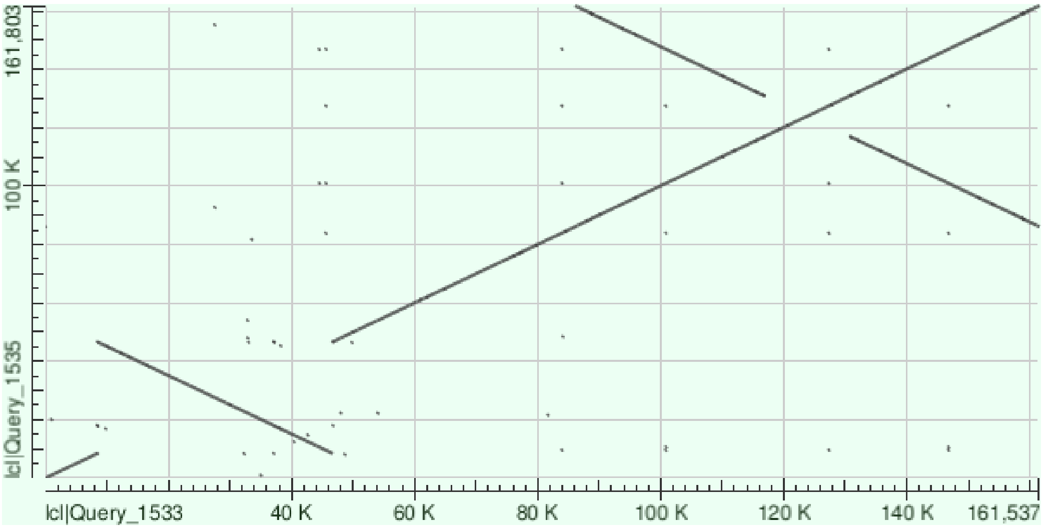

Figure S4. Alignment of the cpDNAs of *T. esculentum* and *T. fassoglense* revealed a 38314 bp long inversion in the LSC region. The *T. esculentum* reference cpDNA was aligned with the *T. fassoglense* cp genome (NC_037767.1) available in NCBI GeneBank using Blastn and shown as a dot plot. The *x*-axis shows the coordinates of the reference cpDNA of *T. esculentum*. The *y*-axis represents the cpDNA of *T. fassoglense*.
